# Characteristics and Outcomes of Second Primary Multiple Myeloma in Adult Cancer Survivors: A Population‐Based Cohort Study

**DOI:** 10.1002/cam4.71393

**Published:** 2025-11-20

**Authors:** Nannan Li, Jiangping Zeng, Zhixiang Jia, Jinyuan Lu, Yuting Ma, Guangming Wang, Aibin Liang, Wenjun Zhang

**Affiliations:** ^1^ Tongji University School of Medicine Shanghai China; ^2^ Department of Hematology, Tongji Hospital Tongji University School of Medicine Shanghai China; ^3^ Department of Endocrinology and Metabolism, Suzhou Dushu Lake Hospital, The Fourth Affiliated Hospital of Soochow University Medical Center of Soochow University Suzhou Jiangsu China; ^4^ Department of Hematology The First Affiliated Hospital of Jinzhou Medical University Jinzhou China; ^5^ Stem Cell Research Center, East Hospital Tongji University School of Medicine Shanghai China

**Keywords:** all‐cause mortality, first primary multiple myeloma, MM‐specific mortality, prior primary malignancy, second primary multiple myeloma, SEER, treatment modalities

## Abstract

**Background:**

This study aimed to investigate characteristics and outcomes of second primary multiple myeloma (2‐MM) stratified by prior primary malignancies (PPMs), and further explore the effect of PPM treatment modalities on multiple myeloma (MM) prognosis in the United States (US) population.

**Methods:**

The retrospective study was analyzed based on MM patients in the Surveillance, Epidemiology, and End Results (SEER) database (2000–2021). Patients with 2‐MM, defined as MM diagnosed ≥ 6 months after a PPM, were stratified into 25 subgroups based on PPM categories. Univariable and multivariable competing risk regression models were constructed to estimate MM‐specific mortality. The Kaplan–Meier method and a multivariable Cox regression model were conducted to analyze all‐cause mortality.

**Result:**

We identified 74,932 patients with first primary multiple myeloma (1‐MM) and 6465 patients with 2‐MM. Compared to the 1‐MM cohort, the 2‐MM cohort exhibited a higher proportion of males, older age at diagnosis, and greater representation of the White race. The multivariable analysis demonstrated a reduced risk of MM‐specific mortality in 2‐MM against 1‐MM. PPMs of soft tissue (including heart), melanoma of the skin, corpus uteri, prostate, kidney and renal pelvis, and lymphoma, showed protective effects on MM‐specific mortality in 2‐MM. Notably, many PPMs that developed into 2‐MM exhibited elevated all‐cause mortality risks in multivariable analysis. Additionally, chemotherapy for prior lymphoma was associated with an increased all‐cause mortality risk in 2‐MM patients, whereas, radiotherapy for prior breast cancer and surgery for prior prostate cancer were linked to a decreased risk.

**Conclusion:**

Compared to 1‐MM, 2‐MM exhibited distinct characteristics and survival outcomes in adults, which were associated with PPM types and their treatment modalities. These findings provide critical insights for risk stratification and survivorship care in US adults.

## Graphical abstract

By leveraging SEER registry data (2000–2021) with rigorous competing risk analysis, we found that second primary multiple myeloma, considered as a vital prognostic factor, exhibited distinct characteristics and survival outcomes compared to first primary multiple myeloma in US adults, which were associated with prior primary malignancy types and their treatment modalities.

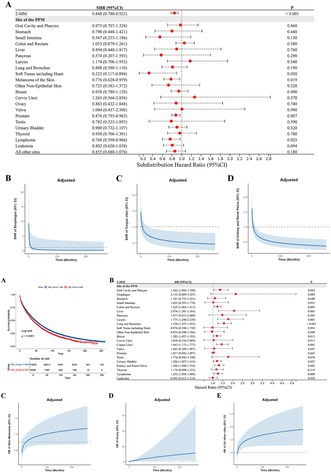

## 1 Introduction

Multiple myeloma (MM) is an incurable dysplasia of clonal plasma cells, which is the second most common hematological malignancy in adults and ultimately causes multiple end‐organ dysfunctions, such as renal insufficiency and bone destruction [1, 2, 3]. Advancements in cancer care have lengthened the cancer survivorship duration and expanded the population of cancer survivors [4]. Nevertheless, cancer survivors may face a growing risk of developing second primary malignancies, which now account for approximately 17% of all incident cancers reported annually to the Surveillance, Epidemiology, and End Results (SEER) program, posing an increasing clinical concern [4, 5]. Similarly, second primary multiple myeloma (2‐MM) should draw clinicians' attention as it accounts for a notable proportion of MM cases [6, 7]. However, there are limited studies regarding 2‐MM.

A previous study by Wang et al. reported that 2‐MM exhibits a higher prevalence of *RB1* gene deletion (13q14) at initial diagnosis compared to those with only primary MM [8]. Furthermore, studies have consistently demonstrated that a history of prior malignancy is associated with poorer survival outcomes in MM patients [7, 9, 10]. These findings suggest that 2‐MM poses a distinct clinical dilemma, likely mediated by host‐related factors (genetic and non‐genetic), as well as disease and therapeutic exposures [7, 11]. However, critical research gaps remain unaddressed in understanding how different prior primary malignancies (PPMs) and their treatment modalities influence the prognosis of 2‐MM, particularly when accounting for confounding factors and competing risks.

In our study, 2‐MM denotes patients diagnosed with MM as a second primary malignancy at least 6 months after a PPM, a latency imposed to distinguish it carefully from recurrent disease. We aimed to investigate the characteristics and outcomes of 2‐MM stratified by PPMs, and to explore the potential effect of PPM treatment modalities on post‐MM prognosis in the United States (US) adult population. MM‐specific mortality was analyzed as the primary endpoint, with all‐cause mortality evaluated as a secondary endpoint.

## 2 Materials and Methods

### 2.1 Data Source and Study Population

This retrospective study analyzed MM patients classified according to the International Classification of Diseases for Oncology, Third Edition (ICD‐O‐3) code 9732 from the SEER database (2000–2021; 17 registries). As illustrated in Figure S1, inclusion and exclusion criteria were applied to ensure accurate case identification. This study used deidentified SEER data under institutional review board (IRB) exemption protocols, requiring no patient consent and complying with international ethics standards (Declaration of Helsinki). Notably, plasma cell leukemia transitioned from ICD‐O‐3 code 9733 (2000–2009) to 9732 post‐2010. Its limited representation (under 1% of cases) [12] and statistical adjustment for diagnosis year minimized classification bias. Additionally, to distinguish 1‐MM from 2‐MM, patients with prior or subsequent plasma cell neoplasms (codes 9731–9734) were excluded to prevent misclassification of potential metastases. In our cohort, 1‐MM refers to individuals for whom MM was the first primary malignancy, whereas 2‐MM denotes patients diagnosed with MM as a second primary malignancy following a PPM, with a mandatory latency period of at least 6 months between diagnoses. The 2‐MM cohort was stratified into 25 subgroups based on PPM categories aligned with the Site Recode ICD‐O‐3/WHO 2008 Definition. To mitigate confounding from post‐MM malignancies, our primary analysis focused on comparing 1‐MM patients (one primary only) with 2‐MM patients without subsequent malignancies. Consistency was confirmed via sensitivity analyses incorporating all MM patients regardless of subsequent malignancies (Figures S5 and S6; Table S2).

### 2.2 Outcomes and Characteristics

MM‐specific mortality (the primary outcome) was calculated from the date of MM diagnosis to death attributed to MM. All‐cause mortality (the secondary outcome) spanned from the date of MM diagnosis to death from any cause. The characteristics related to MM included sex (female vs. male), age at initial diagnosis (18–54, 55–64, 65–74, and 75–90+ years), race (White, Black, Asian or Pacific Islander, and American Indian/Alaska Native), marital status at initial diagnosis (partnered, previously partnered, single, and unknown), and year of diagnosis (2000–2009, 2010–2016, and 2017–2021). Tumor‐directed treatment modalities encompassed chemotherapy (Yes vs. No/Unknown), radiotherapy (Yes vs. No/Unknown), and surgery (Yes vs. No/Unknown) for MM and PPMs.

### 2.3 Statistical Analysis

Continuous variables were expressed as median (interquartile range), and categorical variables as percentages. Comparisons of continuous variables between cohorts were performed using the Mann–Whitney *U* test. Categorical variables across cohorts were compared via chi‐square tests with Bonferroni correction. For MM‐specific mortality, subdistribution hazard ratios (SHRs) and cumulative incidence curves were derived using the Fine–Gray competing risk regression model. Gray's test was employed to compare cumulative incidence across groups. The 1:5 propensity‐score matched analysis was conducted in 2‐MM cohorts (combined or PPM‐stratified subgroups), limiting the analysis to patients with only primary MM. Additionally, all variables were integrated into a random survival forests (RSF) model to assess variable importance (alpha = 0.01). For all‐cause mortality, all analyses were conducted via Kaplan–Meier method and multivariable Cox regression models. In the analysis of competing risk and Cox regression models, the proportional hazards assumption was assessed using Schoenfeld residual tests. For certain PPMs, variables that do not satisfy the assumption, we created time‐dependent covariates that included PPM status by time. All analyses were performed using SPSS 20.0 and R software (version 4.2.2), with *p* < 0.05 considered statistically significant.

## 3 Results

### 3.1 Patient Characteristics

The study identified 74,932 1‐MM and 6465 2‐MM patients, with significant intercohort differences observed in baseline characteristics (Table 1). Specifically, the 2‐MM cohort was older than the 1‐MM cohort (73.0 vs. 67.0 years, respectively), consisted of more men (66.5% vs. 54.3%), White individuals (75.7% vs. 73.0%), and partnered patients (62.2% vs. 57.0%), and had a higher proportion of recent diagnoses (41.6% vs. 30.2% diagnosed in 2017–2021). Lower proportions of 2‐MM patients received chemotherapy (59.6% vs. 64.8%), radiotherapy (14.8% vs. 18.1%) and surgical intervention (0.0% vs. 0.1%) compared to 1‐MM. As shown in Table S1, among the 2‐MM cohort, PPMs of prostate, breast, colon and rectum, skin of the melanoma, urinary bladder, kidney and renal pelvis, and lymphoma represented the most frequent diagnoses. After excluding sex‐specific PPMs, several PPMs that developed into 2‐MM demonstrated significantly higher male predominance compared to 1‐MM. Conversely, thyroid cancer‐associated 2‐MM showed reduced male predominance. Furthermore, multiple PPMs were associated with a lower likelihood of 2‐MM onset at younger ages (< 65 years).

**TABLE 1:** Baseline characteristics of the whole multiple myeloma cohort.

Characteristic	1‐MM (*n* = 74,932)	2‐MM (*n* = 6465)
Male sex^a^	54.3%	66.5%
Age by category, years		
18–54^a^	16.9%	3.8%
55–64^a^	25.0%	14.5%
65–74^a^	30.0%	36.6%
75–90+^a^	28.0%	45.0%
Age, years^a^	67.0 (58.0, 76.0)	73.0 (67.0, 80.0)
Race		
White^a^	73.0%	75.7%
Black^b^	19.8%	19.4%
Asian or Pacific Islander^a^	6.6%	4.4%
American Indian/Alaska Native^b^	0.6%	0.5%
Marital status		
Partnered^a^	57.0%	62.2%
Previously partnered^a^	22.9%	21.7%
Single^a^	13.9%	9.2%
Unknown^b^	6.3%	6.8%
Diagnosis interval		
2000–2009^a^	36.0%	18.3%
2010–2016^a^	33.8%	40.1%
2017–2021^a^	30.2%	41.6%
Chemotherapy for MM^a^	64.8%	59.6%
Radiotherapy for MM^a^	18.1%	14.8%
Surgery for MM^a^	0.1%	0.0%
Chemotherapy for PPM	—	14.6%
Radiotherapy for PPM	—	30.8%
Surgery for PPM	—	65.4%
Latency period, months	—	65.0 (30.0, 114.0)
Follow‐up, months^a^	30.0 (9.0, 66.0)	25.0 (8.0, 53.0)

*Note:* Latency period denotes an interval between the diagnoses of prior primary malignancy and 2‐MM.

Abbreviations: 1‐MM, first primary multiple myeloma; 2‐MM, second primary multiple myeloma; MM, multiple myeloma.

^a^ *p* value < 0.05.

^b^ *p* value ≥ 0.05.

### 3.2 Univariable and Multivariable Analyses of MM‐Specific Mortality

**FIGURE 1:**
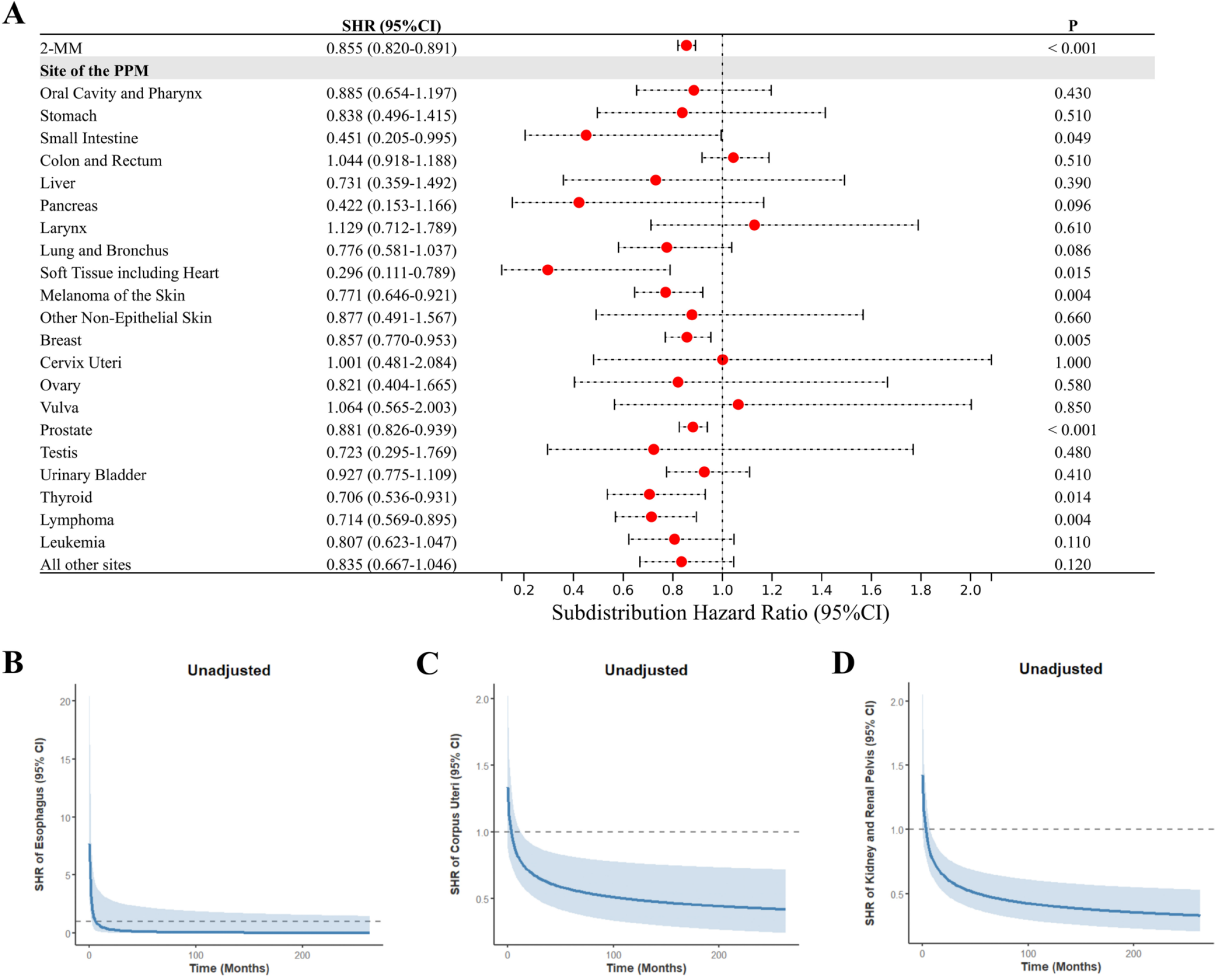
Univariable competing risk regression analysis of MM‐specific mortality. This figure illustrates unadjusted SHRs for MM‐specific mortality (with 1‐MM as reference). (A) SHRs for 2‐MM (combined and PPM‐stratified subgroups). Time‐dependent SHRs for PPMs of (B) esophagus, (C) corpus uteri, and (D) kidney and renal pelvis. 1‐MM, first primary multiple myeloma; 2‐MM, second primary multiple myeloma; CI, confidence interval; MM, multiple myeloma; PPMs, prior primary malignancies; SHR, subdistribution hazard ratio.

In univariable analyses, cumulative incidence of MM‐specific mortality was compared across categories of MM (1‐MM vs. 2‐MM), sex, age at diagnosis, race, marital status at diagnosis, year of MM diagnosis, and treatment modalities (chemotherapy, radiotherapy, and surgery) for MM and PPMs. As detailed in Figure S2, all the analyzed variables except sex were significantly associated with MM‐specific mortality risk. The 2‐MM cohort exhibited significantly lower cumulative incidence of MM‐specific mortality than the 1‐MM cohort, with an unadjusted SHR of 0.855 (95% confidence interval [CI], 0.820–0.891, Figure 1A). In subgroup analyses presented in Figure 1, the univariable competing risk model revealed constant reductions in MM‐specific mortality risk association with small intestine cancer (SHR: 0.451; 95% CI, 0.205–0.995), soft tissue sarcomas including cardiac (SHR: 0.296; 95% CI, 0.111–0.789), melanoma of the skin (SHR: 0.771; 95% CI, 0.646–0.921), breast cancer (SHR: 0.857; 95% CI, 0.770–0.953), prostate cancer (SHR: 0.881; 95% CI, 0.826–0.939), thyroid cancer (SHR: 0.706; 95% CI, 0.536–0.931), and lymphoma (SHR: 0.714; 95% CI, 0.569–0.895). Notably, 2‐MM patients with prior corpus uteri cancer and those with prior kidney and renal pelvis cancers both demonstrated time‐dependent reductions in MM‐specific mortality risk, whereas those with prior esophageal cancer exhibited a transient early elevation in MM‐specific mortality risk confined to the first 2 months post‐diagnosis.

**FIGURE 2:**
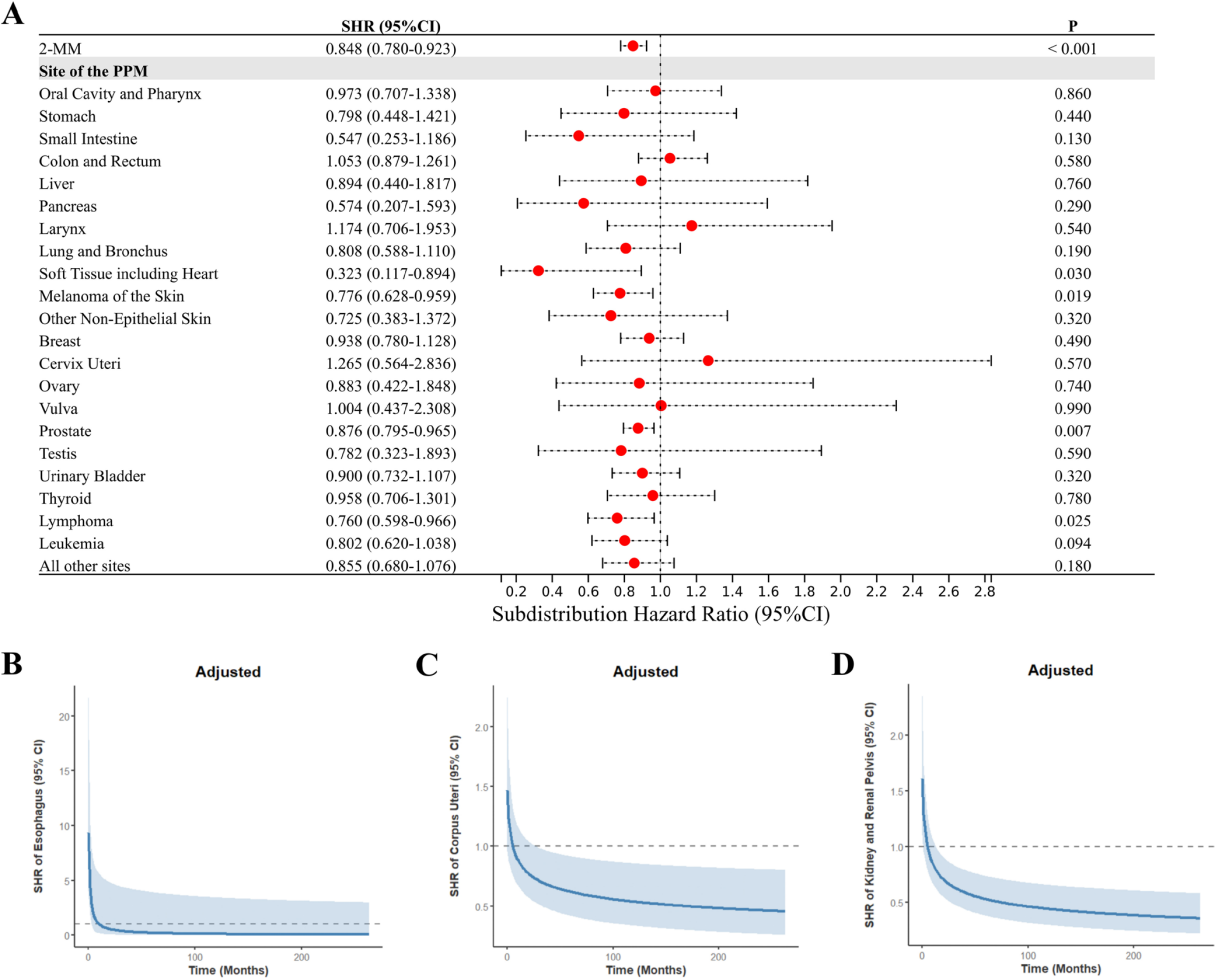
Multivariable competing risk regression analysis of MM‐specific mortality. This figure depicts MM‐specific mortality (with 1‐MM as reference), adjusted for sex, age at diagnosis, race, marital status at diagnosis, year of MM diagnosis, and treatment modalities (chemotherapy, radiotherapy, and surgery) for MM and PPMs. (A) SHRs for 2‐MM (combined and PPM‐stratified subgroups). Time‐dependent SHRs for PPMs of (B) esophagus, (C) corpus uteri, and (D) kidney and renal pelvis. 1‐MM, first primary multiple myeloma; 2‐MM, second primary multiple myeloma; CI, confidence interval; MM, multiple myeloma; PPMs, prior primary malignancies; SHR, subdistribution hazard ratio.

As shown in Figure 2, a multivariable competing risk regression analysis demonstrated that 2‐MM exerted an independent protective role, with an adjusted SHR of 0.848 (95% CI, 0.780–0.923). Specifically, certain PPMs remained associated with reduced MM‐specific mortality risk. These included soft tissue sarcomas (including cardiac) (adjusted SHR: 0.323; 95% CI, 0.117–0.894), melanoma of the skin (adjusted SHR: 0.776; 95% CI, 0.628–0.959), prostate cancer (adjusted SHR: 0.876; 95% CI, 0.795–0.965), and lymphoma (adjusted SHR: 0.760; 95% CI, 0.598–0.966). Additionally, two PPM categories (corpus uteri, and kidney and renal pelvis) exhibited time‐dependent reductions in MM‐specific mortality risk, with adjusted SHRs demonstrating statistically significant declines over time. However, 2‐MM patients with prior kidney and renal pelvis cancers and those with prior esophageal cancer both exhibited a transient early elevation in MM‐specific mortality risk, as detailed in Figure 2. Notably, the reduced MM‐specific mortality risks for the 2‐MM cohorts (combined and specific subgroups stratified by PPMs) were robustly confirmed by propensity score‐matched analysis (Figure S3). Additionally, variable importance was ranked based on a RSF model to predict MM‐specific mortality risk. As shown in Figure S4, the MM group (1‐MM vs. 2‐MM) ranked third in importance, trailing only diagnosis age and year.

Furthermore, the multivariable competing risk regression model was employed to assess the individual effects of PPM treatment modalities (chemotherapy, radiotherapy, and surgery) on MM‐specific mortality across the 2‐MM cohorts (combined and common subgroups stratified by PPMs), while no significant associations were observed (Table 2).

**TABLE 2:** Multivariable analyses of MM‐specific and all‐cause mortality associated with PPM treatment modalities in 2‐MM cohorts.

2‐MM	MM‐specific mortality			All‐cause mortality		
	Adjusted SHR (95% CI)			Adjusted HR (95% CI)		
	Chemotherapy	Radiotherapy	Surgery	Chemotherapy	Radiotherapy	Surgery
Combined PPMs (*n* = 6465)	1.039 (0.918–1.176)	1.027 (0.938–1.125)	0.994 (0.906–1.091)	1.266 (1.153–1.391)^a^	0.928 (0.863–0.998)^a^	0.870 (0.808–0.937)^a^
Prostate (*n* = 2474)	—	1.005 (0.865–1.166)	0.971 (0.833–1.131)	—	0.936 (0.832–1.053)	0.749 (0.659–0.851)^a^
Breast (*n* = 916)	0.935 (0.730–1.197)	0.943 (0.758–1.174)	—	1.000 (0.816–1.225)	0.742 (0.621–0.885)^a^	—
Colon and rectum (*n* = 550)	1.129 (0.802–1.590)	0.957 (0.611–1.500)	—	1.060 (0.791–1.421)	1.034 (0.703–1.519)	—
Urinary bladder (*n* = 330)	1.246 (0.701–2.213)	—	—	1.084 (0.686–1.713)	—	—
Lymphoma (*n* = 254)	1.493 (0.946–2.355)	0.853 (0.485–1.501)	0.712 (0.428–1.186)	1.426 (1.014–2.006)^a^	0.883 (0.585–1.334)	1.096 (0.757–1.588)
Corpus uteri (*n* = 199)	0.220 (0.047–1.025)	1.166 (0.557–2.442)	—	0.592 (0.291–1.207)	1.389 (0.833–2.317)	—

*Note:* MM‐specific and all‐cause mortality for the 2‐MM cohorts (combined or PPM‐stratified subgroups) were assessed in multivariable models, adjusted for sex, age at diagnosis, race, marital status at diagnosis, year of MM diagnosis, and the other tumor‐directed treatment modalities. Specific PPM subgroups were excluded from analyses of particular treatments due to insufficient sample sizes in strata following stratification by PPM treatment modalities.

Abbreviations: 2‐MM, second primary multiple myeloma; CI, confidence interval; HR, hazard ratio; MM, multiple myeloma; PPM, prior primary malignancy; SHR, subdistribution hazard ratio.

^a^ *p* value < 0.05.

### 3.3 Univariable and Multivariable Analyses of All‐Cause Mortality

**FIGURE 3:**
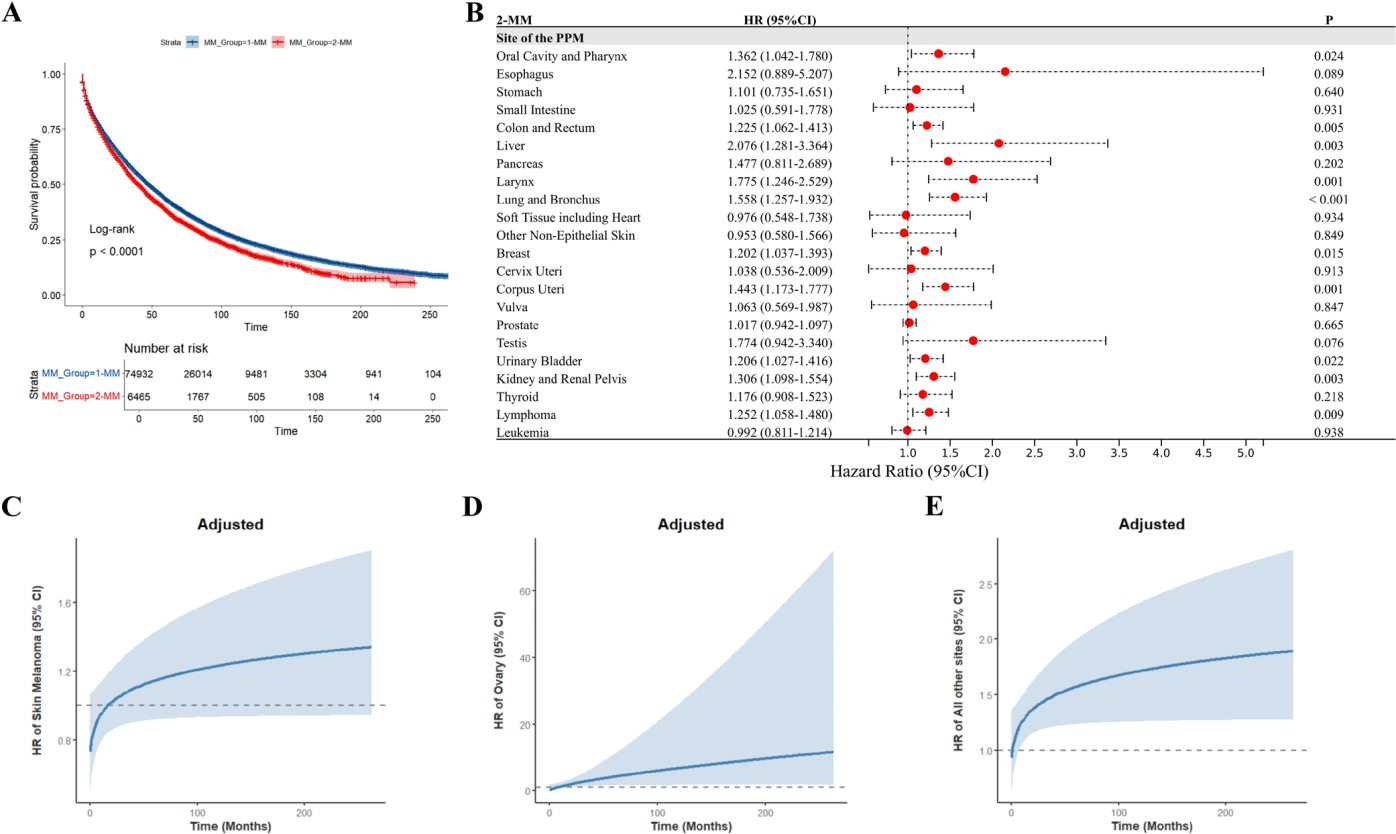
Univariable and multivariable analyses of all‐cause mortality. (A) Kaplan–Meier curves of overall survival for 2‐MM vs. 1‐MM. (B–E) Multivariable Cox regression analysis of all‐cause mortality (with 1‐MM as reference), adjusted for sex, age at diagnosis, race, marital status at diagnosis, year of MM diagnosis, and treatment modalities (chemotherapy, radiotherapy, and surgery) for MM and PPMs. (B) HRs for 2‐MM (combined and PPM‐stratified subgroups); time‐dependent HRs for PPMs of (C) melanoma of the skin, (D) ovary, and (E) all other sites. 1‐MM, first primary multiple myeloma; 2‐MM, second primary multiple myeloma; CI, confidence interval; HR, hazard ratio; MM, multiple myeloma; PPMs, prior primary malignancies.

The 2‐MM cohort demonstrated shorter median OS than 1‐MM (41 vs. 48 months), with Kaplan–Meier analysis confirming inferior survival outcomes (Figure 3A). Multivariable Cox regression analysis was performed to determine adjusted hazard ratios (HRs) for evaluating the role of distinct PPM subgroups in 2‐MM as potential risk factors for all‐cause mortality (Figure 3B–E). This revealed that multiple PPM subgroups were associated with increased risks of all‐cause mortality, including cancers of the oral cavity and pharynx, colon and rectum, liver, larynx, lung and bronchus, breast, corpus uteri, ovary, urinary bladder, kidney and renal pelvis, lymphoma, and all other sites. Liver cancer showed the highest mortality risk (adjusted HR: 2.076; 95% CI, 1.281–3.364).

Additionally, multivariable Cox regression analyses were conducted to assess the impact of PPM treatment modalities as potential risk factors for all‐cause mortality in the 2‐MM cohorts (combined and common subgroups stratified by PPMs), with results presented in Table 2. Chemotherapy for combined PPMs was associated with an elevated risk of all‐cause mortality (adjusted HR: 1.266; 95% CI, 1.153–1.391), as was lymphoma (adjusted HR: 1.426; 95% CI, 1.014–2.006). However, no significant associations were found for breast, colon and rectal, urinary bladder, and corpus uteri cancers. Radiotherapy‐related analyses demonstrated reduced all‐cause mortality risk for combined PPMs (adjusted HR: 0.928; 95% CI, 0.863–0.998) and breast cancer (adjusted HR: 0.742; 95% CI, 0.621–0.885), whereas no statistically significant associations were identified in prostate, colon and rectal, corpus uteri cancers, and lymphoma. Similarly, surgical intervention was associated with significantly lower all‐cause mortality risk for combined PPMs (adjusted HR: 0.870; 95% CI, 0.808–0.937) and prostate cancer (adjusted HR: 0.749; 95% CI, 0.659–0.851), while no significant effect was observed for lymphoma.

## 4 Discussion

In our study of US adults, we found that 2‐MM patients exhibited an independently lower risk of MM‐specific mortality compared to those with 1‐MM. Specifically, PPMs of soft tissue (including heart), melanoma of the skin, corpus uteri, prostate, kidney and renal pelvis, and lymphoma were independently associated with a decreased risk of MM‐specific mortality. Furthermore, our analyses demonstrated that 2‐MM was associated with elevated all‐cause mortality risk relative to 1‐MM, with multiple PPMs independently linked to increased all‐cause mortality. In addition, PPM treatment modalities exhibited discordant relationships with all‐cause mortality risk in 2‐MM patients.

The common PPMs in our 2‐MM cohort were generally aligned with Engelhardt et al.'s study [9] and Jonsdottir et al.'s study [7]. Notably, the demographic characteristics observed in the 2‐MM cohort can be linked to PPMs, considering that these types of MM developed subsequently to the prior malignancies [13, 14]. The 2‐MM cohort demonstrated distinct clinical characteristics versus 1‐MM, including a higher proportion of males, older age at diagnosis, and greater representation of the White race (Table 1). Published data on these differences remain limited. Although two prior studies reported concordant results regarding the diagnosis age, there are conflicting results in the male proportion, which may be related to population selection [7, 9].

We further found a significantly lower MM‐specific mortality risk in 2‐MM patients compared to 1‐MM. After stratifying by PPMs, the survival advantage remained statistically significant for PPMs of soft tissue (including heart), melanoma of the skin, corpus uteri, prostate, kidney and renal pelvis, and lymphoma, while 2‐MM proved non‐inferior in all remaining PPM strata. Since clinical and experimental data regarding the correlation between 2‐MM and these PPMs remain limited, the exact mechanism for the observed reduction in MM‐specific mortality is unclear. However, there are still studies that provided support for our findings.

Patients with a PPM usually undergo comprehensive health assessments and monitoring. This would facilitate earlier detection and timely intervention of 2‐MM, thereby leading to reduced MM‐specific mortality [15]. The treatments for PPMs, such as cyclophosphamide, venetoclax, and zoledronic acid, could affect MM‐specific prognosis [16, 17, 18]. The National Comprehensive Cancer Network (NCCN) MM Panel has listed cyclophosphamide‐containing combination regimens among the other recommended options for relapsed or refractory MM patients who have received one to three prior lines of therapy [16]. Meanwhile, clinical studies have reported that cyclophosphamide, when given at a high dose or in a hyperfractionated schedule, can achieve rapid disease control in MM, leading the panel to include it as an option for relapsed/refractory MM [16, 19, 20]. Additionally, venetoclax‐containing regimens have documented efficacy and safety in MM patients with t(11;14) in several prospective trials [21, 22]. Zoledronic acid, a bone‐targeted agent approved for the treatment of multiple myeloma as well as documented bone metastases from solid tumors, was reported to improve antimyeloma effect and extend survival [17, 23]. However, we observed no significant association between PPM treatment modalities (chemotherapy, radiotherapy, and surgery) and MM‐specific mortality. This finding parallels Feng et al.'s study on second primary differentiated thyroid cancer in the SEER database [24]. Given that detailed data regarding PPM treatment regimens, including treatment timing/dosage, anatomical sites of radiotherapy/surgery, and other therapies (e.g., immunotherapy, targeted and endocrine therapy), were absent in the SEER database, our further research was limited on this issue. Moreover, we considered whether genetic factors may contribute to these observed risk changes [7, 9, 14, 25]. Several genetic alterations shared between MM and other malignancies, such as *BRAF* V600E, *BRCA1/2*, and *IRF4* mutations, have been suggested to correlate with a potentially more favorable MM prognosis [26, 27, 28, 29, 30, 31, 32], for which further investigation is warranted. Overall, the surveillance and treatments for PPMs, and potential genetic mutations between 2‐MM and PPMs, may play a role in this regard.

Despite the survival advantage in MM‐specific mortality observed in 2‐MM patients, their all‐cause mortality risk was elevated, with subgroup analyses revealing statistically significant associations for multiple PPMs. Interestingly, this finding aligns with Jonsdottir et al.'s report of a detrimental impact of prior malignancies on OS in MM patients [7].

Additionally, we identified discordant associations between PPM treatment modalities and all‐cause mortality in 2‐MM cohorts. For chemotherapy, increased risk was observed only in lymphoma and combined PPMs, whereas no significant associations were detected for solid tumor. Notably, a study of 15,521 solid tumor and 2537 hematological malignancy patients demonstrated that hematological malignancy patients faced higher risks of post‐chemotherapy thrombocytopenia compared to solid tumor patients [33], suggesting that chemotherapy for prior lymphoma may exert more pronounced adverse effects on MM survival than that for solid tumor, although this hypothesis requires validation in future studies. In contrast, while evaluating the impact of surgery across prostate cancer, lymphoma, and combined PPMs, risk reductions were observed only for prostate cancer and combined PPMs. Evidence indicates that radical prostatectomy, the standard treatment modality for prostate cancer, effectively reduces late recurrence risk and associated mortality [34, 35, 36], implying that optimal surgery management of prior prostate cancer may confer survival advantages in 2‐MM. For radiotherapy, risk reductions were confined to breast cancer and combined PPMs, with no significant associations observed in the other analyzed subgroups stratified by PPMs. In contrast to our findings, a prior SEER database study of primary breast cancer (1988–2013) reported that radiotherapy reduced the risk of developing subsequent MM but had no impact on its 15‐year OS [37]. The survival impact of breast cancer radiotherapy primarily manifested as reduced breast cancer‐specific mortality and elevated non‐breast cancer mortality (e.g., lung and esophageal cancers, cardiotoxicity, thromboembolism), with radiotherapy‐related risks increasing with organ‐specific radiation dose escalation [38]. This suggests that the improved survival outcomes linked to breast cancer radiotherapy in our 2‐MM cohort may be attributable to advancements in radiotherapy techniques and reduced organ‐specific doses [38], particularly given the more recent diagnostic timeframe for primary breast cancer in our study.

These findings underscore that prognostic evaluation of 2‐MM patients should not be confined to the assessment of MM condition alone; PPM types and associated treatment modalities should also be integrated into clinical considerations. Given the favorable MM‐specific prognosis observed in 2‐MM patients with protective PPMs (e.g., prostate cancer), de‐escalated treatment strategies may be warranted for these subgroups. Additionally, intensified multidisciplinary surveillance and management should be recommended for 2‐MM patients exhibiting poorer OS profiles.

There are several limitations in our study. First, in line with previous retrospective studies, potential selection biases were introduced into the results due to the absence of detailed information on clinical staging, cytogenetic abnormalities, and therapeutic regimens. Nevertheless, our analyses were conducted on a large, multicenter US population, and all available confounders were adjusted to mitigate biases from non‐randomized factors. Second, specific information about the regimens and complications of PPM treatment modalities may provide access to a more comprehensive analysis of relationships between PPM treatment modalities and MM survival outcomes, but such data are not available in the SEER database. Third, limited data were available for rare PPMs, such as esophageal, pancreatic, cervical, and ovarian cancers, necessitating caution in interpreting these findings. Fourth, as the SEER database captures only the subset of US residents whose data are formally entered into the registry, the broader generalizability and external validity of our findings require further confirmation across diverse geographic settings.

However, our study also exhibits notable strengths. First, we provide novel insights into the distinct clinical and survival patterns of 2‐MM compared to 1‐MM, further evaluating the prognostic effects of PPM types and their treatment modalities using a large, population‐based dataset with long‐term follow‐up. Second, whereas prior studies often inadequately addressed competing mortality risks, our study concurrently assessed both MM‐specific and all‐cause mortality. Third, previous studies often conflated heterogeneous cohorts of multiple primary malignancies. To minimize confounding from subsequent malignancies, we restricted the primary analysis to patients without post‐MM malignancies but included these patients in sensitivity analyses to ensure robustness.

## 5 Conclusions

In summary, 2‐MM demonstrated lower MM‐specific mortality compared to 1‐MM, and PPMs of soft tissue (including heart), melanoma of the skin, corpus uteri, prostate, kidney and renal pelvis, and lymphoma exhibited significantly reduced MM‐specific mortality risk. Notably, multiple PPMs independently and adversely impacted OS prognosis in 2‐MM patients. Additionally, PPM treatment modalities displayed discordant relationships with all‐cause mortality risk in 2‐MM patients. This study elucidates the prognostic implications of PPM types and their treatment modalities in 2‐MM, providing critical insights for personalized survivorship care in US adults.

## Author Contributions

**Nannan Li:** data curation (equal), formal analysis (equal), investigation (equal), methodology (equal), software (lead), validation (equal), visualization (equal), writing – original draft (lead), writing – review and editing (equal). **Jiangping Zeng:** formal analysis (equal), investigation (equal), methodology (equal), validation (equal), visualization (equal), writing – review and editing (equal). **Zhixiang Jia:** investigation (equal), methodology (equal), supervision (equal), validation (equal), writing – review and editing (equal). **Jinyuan Lu:** investigation (equal), methodology (equal), validation (equal), writing – review and editing (equal). **Yuting Ma:** investigation (equal), methodology (equal), validation (equal), writing – review and editing (equal). **Guangming Wang:** investigation (equal), methodology (equal), validation (equal), writing – review and editing (equal). **Aibin Liang:** conceptualization (equal), investigation (equal), methodology (equal), project administration (equal), resources (equal), supervision (equal), validation (equal), writing – review and editing (equal). **Wenjun Zhang:** conceptualization (equal), data curation (equal), funding acquisition (lead), investigation (equal), methodology (equal), project administration (equal), resources (equal), supervision (equal), validation (equal), writing – review and editing (equal).

## Ethics Statement

This study used deidentified SEER data under institutional review board (IRB) exemption protocols, requiring no patient consent and complying with international ethics standards (Declaration of Helsinki).

## Conflicts of Interest

The authors declare no conflicts of interest.

## Supporting information


**Figure S1:** Flowchart of patient data selection for multiple myeloma (MM). 1‐MM, first primary multiple myeloma; 2‐MM, second primary multiple myeloma; 9731, solitary plasmacytoma of bone; 9732, plasma cell myeloma; 9733, plasma cell leukemia; 9734, extraosseous plasmacytoma.
**Figure S2:** Cumulative incidence curves of MM‐specific death analyzed in MM patients by subgroups, treating non‐MM deaths as a competing event. (A) MM group (1‐MM and 2‐MM); (B) sex; (C) age at diagnosis; (D) race; (E) marital status at diagnosis; (F) year of MM diagnosis; (G) chemotherapy, (H) radiotherapy, and (I) surgery for MM; (J) chemotherapy, (K) radiotherapy, and (L) surgery for PPMs. MM, multiple myeloma; 1‐MM, first primary multiple myeloma; 2‐MM, second primary multiple myeloma; PPM, prior primary malignancy.
**Figure S3:** Cumulative incidence curves of MM‐specific mortality between 2‐MM cohorts (combined or subgroups stratified by PPMs) and propensity‐score matched 1‐MM controls, with non‐MM deaths treated as a competing event. (A) Combined vs. 1‐MM controls; (B) soft tissue (including heart) vs. 1‐MM controls; (C) melanoma of the skin vs. 1‐MM controls; (D) lymphoma vs. 1‐MM controls; (E) prostate vs. 1‐MM controls; (F) kidney and renal pelvis vs. 1‐MM controls; (G‐H) corpus uteri vs. 1‐MM controls. MM, multiple myeloma; 1‐MM, first primary multiple myeloma; 2‐MM, second primary multiple myeloma; PPMs, prior primary malignancies.
**Figure S4:** Variable importance for MM‐specific mortality predicted by the Random Survival Forest (RSF) model. (A) parametric and (B) non‐parametric confidence interval methods. Evaluated predictors included: MM group (1‐MM vs. 2‐MM), sex, age at diagnosis (categorical), race, marital status at diagnosis, year of MM diagnosis (categorical), and treatment modalities (chemotherapy/radiotherapy/surgery for MM; chemotherapy/radiotherapy/surgery for PPMs). VIMP, variable importance; MM, multiple myeloma; 1‐MM, first primary multiple myeloma; 2‐MM, second primary multiple myeloma; PPM, prior primary malignancy.
**Figure S5:** Sensitivity analysis of MM‐specific mortality in the study cohort incorporating all MM patients regardless of subsequent malignancies. This figure depicts multivariable competing risk regression analysis of MM‐specific mortality (with 1‐MM as reference), adjusted for sex, age at diagnosis, race, marital status at diagnosis, year of MM diagnosis, and treatment modalities (chemotherapy, radiotherapy, and surgery) for MM and PPMs. (A) SHRs for 2‐MM (combined and PPM‐stratified subgroups); Time‐dependent SHRs for PPMs of (B) esophagus, (C) corpus uteri, and (D) kidney and renal pelvis. MM, multiple myeloma; 1‐MM, first primary multiple myeloma; 2‐MM, second primary multiple myeloma; SHR, subdistribution hazard ratio; CI, confidence interval; PPMs, prior primary malignancies.
**Figure S6:** Sensitivity analysis of all‐cause mortality in the study cohort incorporating all MM patients regardless of subsequent malignancies. (A) Kaplan–Meier curves of overall survival for 2‐MM vs. 1‐MM. (B‐E) Multivariable Cox regression analysis of all‐cause mortality (with 1‐MM as reference), adjusted for sex, age at diagnosis, race, marital status at diagnosis, year of MM diagnosis, and treatment modalities (chemotherapy, radiotherapy, and surgery) for MM and PPMs. (B) HRs for 2‐MM (combined and PPM‐stratified subgroups); Time‐dependent HRs for PPMs of (C) lung and bronchus, (D) melanoma of the skin, and (E) breast. MM, multiple myeloma; 1‐MM, first primary multiple myeloma; 2‐MM, second primary multiple myeloma; HR, hazard ratio; CI, confidence interval; PPMs, prior primary malignancies.
**Table S1:** Clinical characteristics of 2‐MM cohort according to PPMs.
**Table S2:** Sensitivity analysis of MM‐specific and all‐cause mortality for PPM treatment modalities in 2‐MM cohorts.

## Data Availability

The datasets analyzed during the current study are available in the Surveillance, Epidemiology, and End Results (SEER) database repository (www.seer.cancer.gov).
