# Supplementary material for: Characteristics and Outcomes of Second Primary Multiple Myeloma in Adult Cancer Survivors: A Population‐Based Cohort Study
Source: Cancer Med. 2025 Nov 20;14(22):e71393. doi: 10.1002/cam4.71393 (PMC12633652; doi:10.1002/cam4.71393)
Supplement: Supplementary file 1 — Figure S1: Flowchart of patient data selection for multiple myeloma (MM). 1‐MM, first primary multiple myeloma; 2‐MM, second primary multiple myeloma; 9731, solitary plasmacytoma of bone; 9732, plasma cell myeloma; 9733, plasma cell leukemia; 9734, extraosseous plasmacytoma. Figure S2: Cumulative incidence curves of MM‐specific death analyzed in MM patients by subgroups, treating non‐MM deaths as a competing event. (A) MM group (1‐MM and 2‐MM); (B) sex; (C) age at diagnosis; (D) race; (E) marital status at diagnosis; (F) year of MM diagnosis; (G) chemotherapy, (H) radiotherapy, and (I) surgery for MM; (J) chemotherapy, (K) radiotherapy, and (L) surgery for PPMs. MM, multiple myeloma; 1‐MM, first primary multiple myeloma; 2‐MM, second primary multiple myeloma; PPM, prior primary malignancy. Figure S3: Cumulative incidence curves of MM‐specific mortality between 2‐MM cohorts (combined or subgroups stratified by PPMs) and propensity‐score matched 1‐MM controls, with non‐MM deaths treated as a competing event. (A) Combined vs. 1‐MM controls; (B) soft tissue (including heart) vs. 1‐MM controls; (C) melanoma of the skin vs. 1‐MM controls; (D) lymphoma vs. 1‐MM controls; (E) prostate vs. 1‐MM controls; (F) kidney and renal pelvis vs. 1‐MM controls; (G‐H) corpus uteri vs. 1‐MM controls. MM, multiple myeloma; 1‐MM, first primary multiple myeloma; 2‐MM, second primary multiple myeloma; PPMs, prior primary malignancies. Figure S4: Variable importance for MM‐specific mortality predicted by the Random Survival Forest (RSF) model. (A) parametric and (B) non‐parametric confidence interval methods. Evaluated predictors included: MM group (1‐MM vs. 2‐MM), sex, age at diagnosis (categorical), race, marital status at diagnosis, year of MM diagnosis (categorical), and treatment modalities (chemotherapy/radiotherapy/surgery for MM; chemotherapy/radiotherapy/surgery for PPMs). VIMP, variable importance; MM, multiple myeloma; 1‐MM, first primary multiple myeloma; 2‐MM, second primary mult [file CAM4-14-e71393-s001.docx]

**Supplementary Material**

**Supplementary Information**

**PPM category**

The “All other sites” category within prior primary malignancies (PPMs) encompassed the eye and orbit; peritoneum, omentum, and mesentery; Kaposi sarcoma; nose, nasal cavity, and middle ear; brain; bones and joints; retroperitoneum; gallbladder, and ureter, among others.

**Time-dependent SHRs and HRs**

For certain PPMs, variables that does not satisfy the proportional hazards assumption, we created time-dependent covariates that included PPM status by time. Time-dependent subdistribution hazard ratios (SHRs) and hazard ratios (HRs) for these PPMs (with 1-MM as reference) were modelled with x (fixed term) and x × ln (t + 1) (interaction term), where x is exposure variable, and t represents follow-up time from MM diagnosis.

Primary analyses were conducted in MM patients without subsequent malignancies. Univariable competing risks regression analysis of MM-specific mortality is presented in Figure 1B-D. For esophagus, the SHR was 7.677 (*p* < 0.001) for main effects and 0.350 (*p* = 0.012) for the interaction term; for corpus uteri, the SHR was 1.335 (*p* = 0.170) for main effects and 0.813 (*p* = 0.006) for the interaction term; and for kidney and renal pelvis, the SHR was 1.426 (*p* = 0.056) for main effects and 0.769 (*p* < 0.001) for the interaction term. Multivariable competing risk regression analysis of MM-specific mortality is shown in Figure 2B-D. For esophagus, the SHR was 9.369 (*p* < 0.001) for main effects and 0.397 (*p* = 0.017) for the interaction term; for corpus uteri, the SHR was 1.469 (*p* = 0.076) for main effects and 0.810 (*p* = 0.005) for the interaction term; and for kidney and renal pelvis, the SHR was 1.610 (*p* = 0.014) for main effects and 0.762 (*p* < 0.001) for the interaction term. Multivariable Cox regression analysis of all-cause mortality is summarized in Figure 3C-E. For melanoma of the skin, the HR was 0.734 (*p* = 0.105) for main effects and 1.114 (*p* = 0.065) for the interaction term; for ovary, the HR was 0.247 (*p* = 0.173) for main effects and 1.994 (*p* = 0.039) for the interaction term; and for all other sites, the HR was 0.931 (*p* = 0.712) for main effects and 1.136 (*p* = 0.047) for the interaction term.

Sensitivity analyses were performed in the study cohort incorporating all MM patients regardless of subsequent malignancies. Sensitivity analysis of multivariable competing risk regression for MM-specific mortality is shown in Figure S5B-D. For esophagus, the SHR was 9.335 (*p* < 0.001) for main effects and 0.404 (*p* = 0.021) for the interaction term; for corpus uteri, the SHR was 1.504 (*p* = 0.057) for main effects and 0.805 (*p* = 0.003) for the interaction term; and for kidney and renal pelvis, the SHR was 1.628 (*p* = 0.010) for main effects and 0.762 (*p* < 0.001) for the interaction term. Sensitivity analysis of multivariable Cox regression for all-cause mortality is presented in Figure S6C-E. For lung and bronchus, the HR was 0.983 (*p* = 0.939) for main effects and 1.157 (*p* = 0.041) for the interaction term; for melanoma of the skin, the HR was 0.717 (*p* = 0.073) for main effects and 1.121 (*p* = 0.039) for the interaction term; and for breast, the HR was 0.975 (*p* = 0.833) for main effects and 1.078 (*p* = 0.023) for the interaction term.

**Supplementary Figures**

**Supplementary Figure 1**


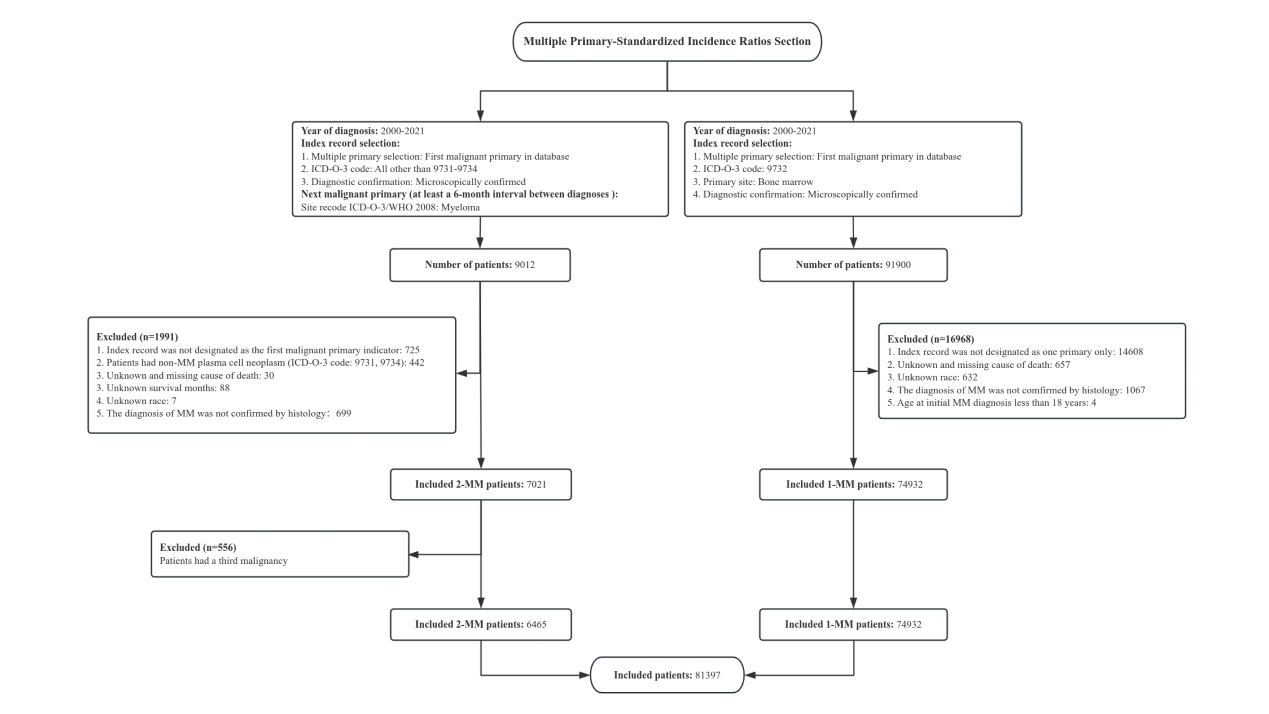


Supplementary Figure 1. Flowchart of patient data selection for multiple myeloma (MM). 1-MM, first primary multiple myeloma; 2-MM, second primary multiple myeloma; 9731, solitary plasmacytoma of bone; 9732, plasma cell myeloma; 9733, plasma cell leukemia; 9734, extraosseous plasmacytoma.

**Supplementary Figure 2**


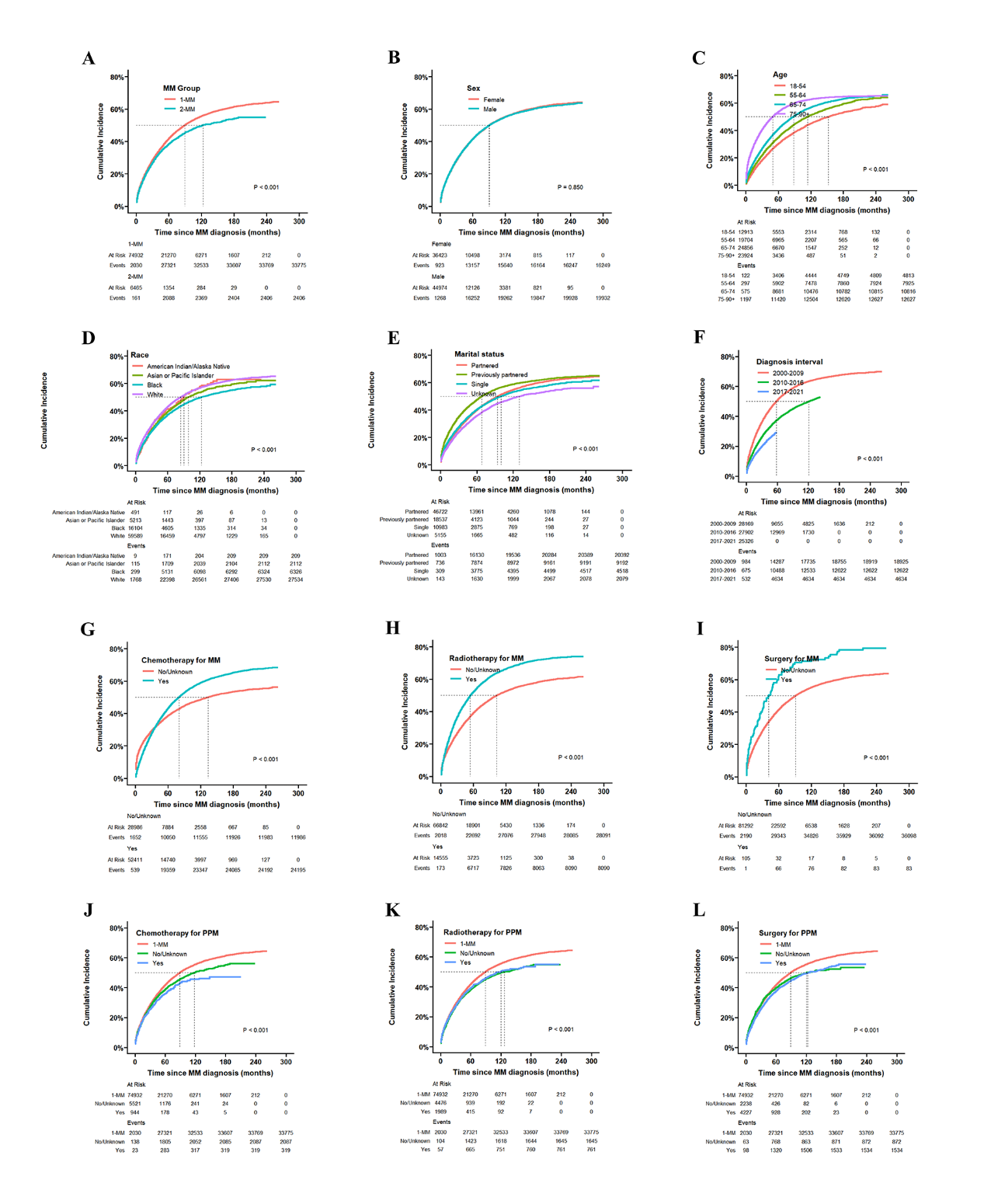


Supplementary Figure 2. Cumulative incidence curves of MM-specific death analyzed in MM patients by subgroups, treating non-MM deaths as a competing event. (A) MM group (1-MM and 2-MM); (B) sex; (C) age at diagnosis; (D) race; (E) marital status at diagnosis; (F) year of MM diagnosis; (G) chemotherapy, (H) radiotherapy, and (I) surgery for MM; (J) chemotherapy, (K) radiotherapy, and (L) surgery for PPMs. MM, multiple myeloma; 1-MM, first primary multiple myeloma; 2-MM, second primary multiple myeloma; PPM, prior primary malignancy.

**Supplementary Figure 3**


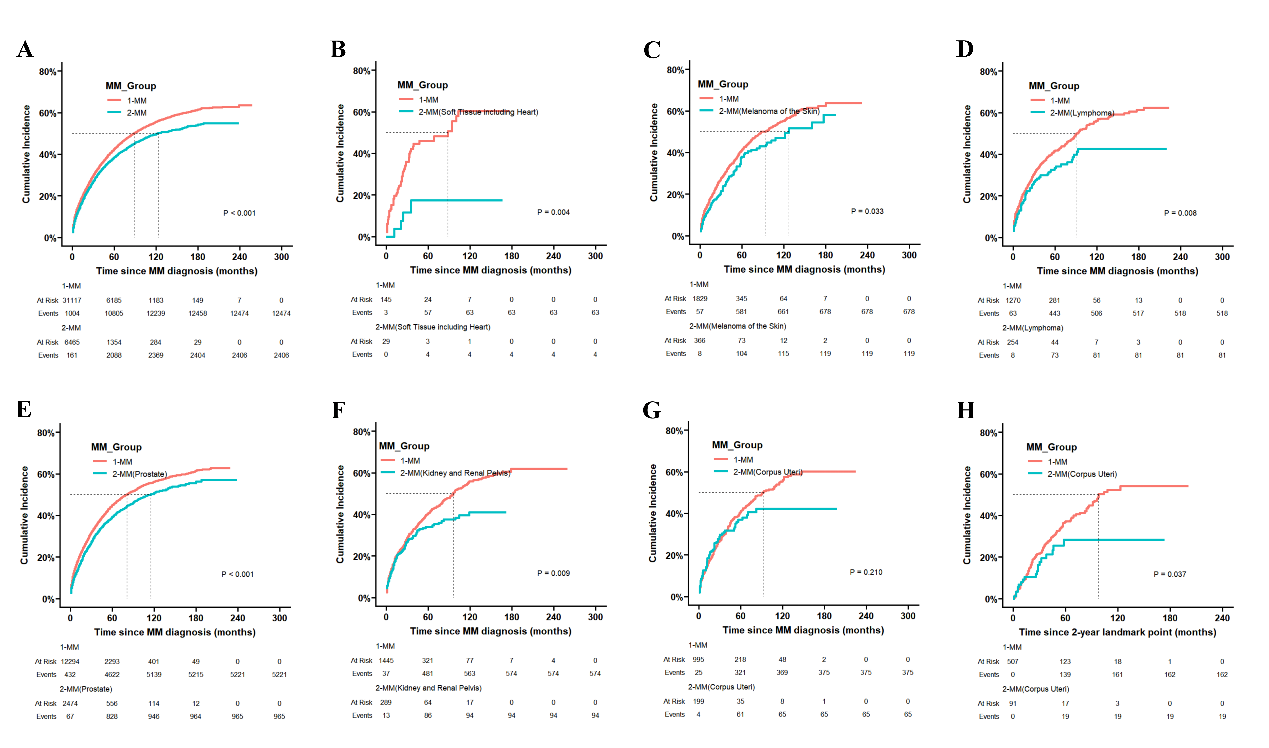


Supplementary Figure 3. Cumulative incidence curves of MM-specific mortality between 2-MM cohorts (combined or subgroups stratified by PPMs) and propensity-score matched 1-MM controls, with non-MM deaths treated as a competing event. (A) Combined vs. 1-MM controls; (B) soft tissue (including heart) vs. 1-MM controls; (C) melanoma of the skin vs. 1-MM controls; (D) lymphoma vs. 1-MM controls; (E) prostate vs. 1-MM controls; (F) kidney and renal pelvis vs. 1-MM controls; (G-H) corpus uteri vs. 1-MM controls. MM, multiple myeloma; 1-MM, first primary multiple myeloma; 2-MM, second primary multiple myeloma; PPMs, prior primary malignancies.

**Supplementary Figure 4**


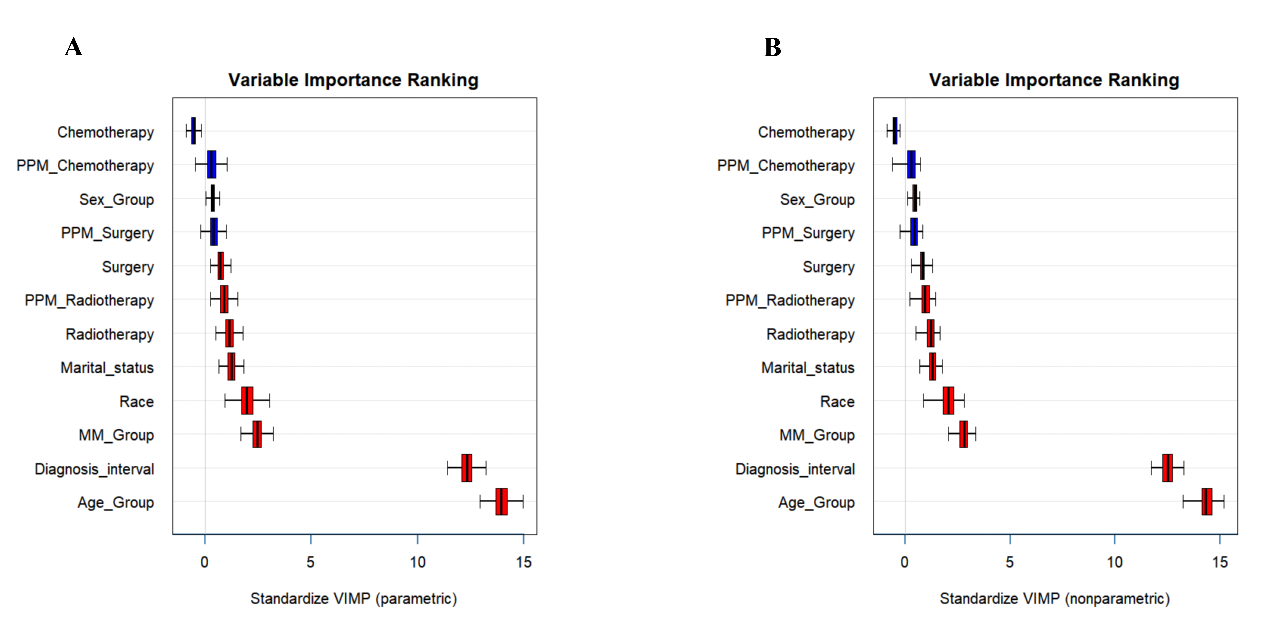


Supplementary Figure 4. Variable importance for MM-specific mortality predicted by the Random Survival Forest (RSF) model. (A) parametric and (B) non-parametric confidence interval methods. Evaluated predictors included: MM group (1-MM vs. 2-MM), sex, age at diagnosis (categorical), race, marital status at diagnosis, year of MM diagnosis (categorical), and treatment modalities (chemotherapy/radiotherapy/surgery for MM; chemotherapy/radiotherapy/surgery for PPMs). VIMP, variable importance; MM, multiple myeloma; 1-MM, first primary multiple myeloma; 2-MM, second primary multiple myeloma; PPM, prior primary malignancy.

**Supplementary Figure 5**


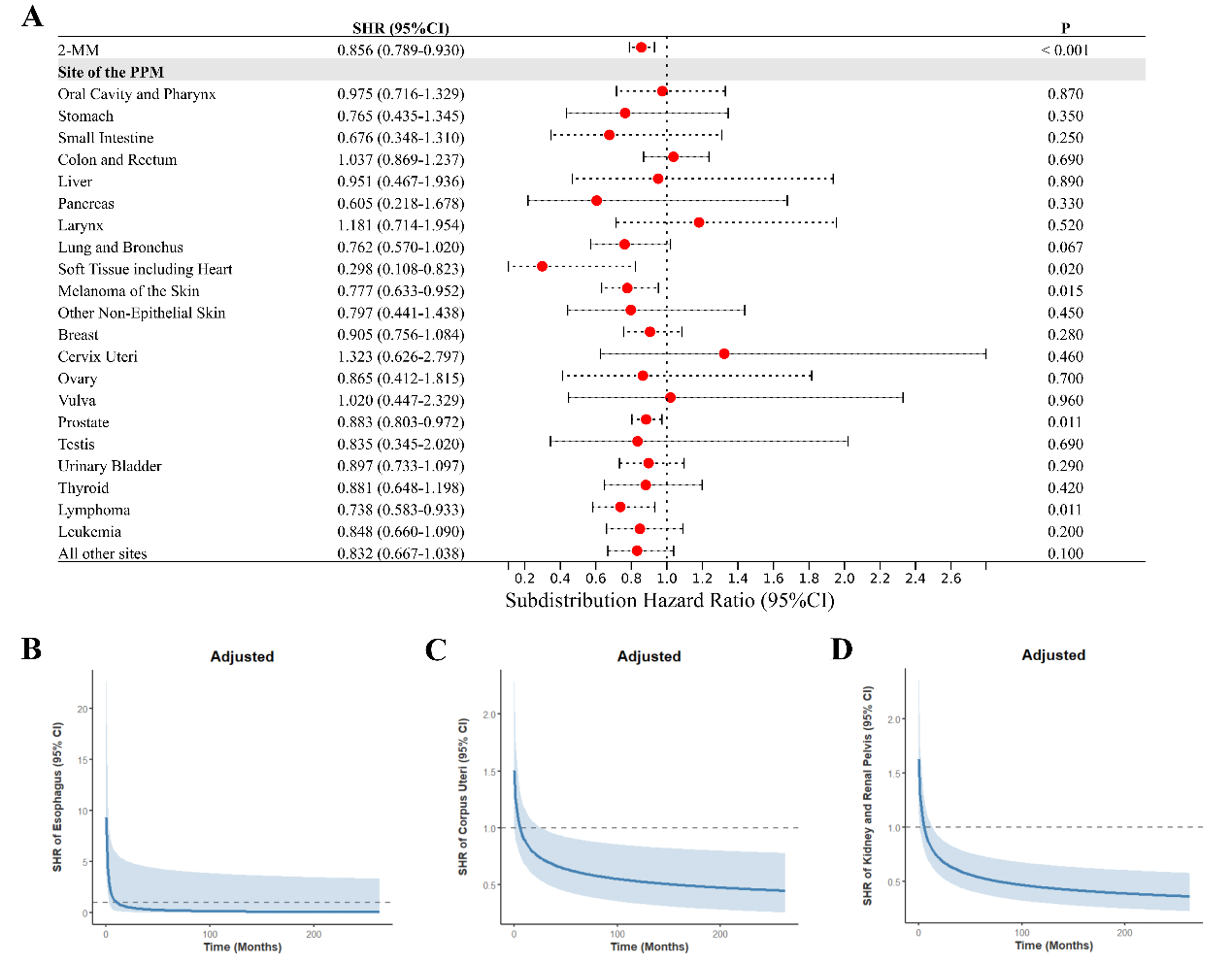


Supplementary Figure 5. Sensitivity analysis of MM-specific mortality in the study cohort incorporating all MM patients regardless of subsequent malignancies. This figure depicts multivariable competing risk regression analysis of MM-specific mortality (with 1-MM as reference), adjusted for sex, age at diagnosis, race, marital status at diagnosis, year of MM diagnosis, and treatment modalities (chemotherapy, radiotherapy, and surgery) for MM and PPMs. (A) SHRs for 2-MM (combined and PPM-stratified subgroups); Time-dependent SHRs for PPMs of (B) esophagus, (C) corpus uteri, and (D) kidney and renal pelvis. MM, multiple myeloma; 1-MM, first primary multiple myeloma; 2-MM, second primary multiple myeloma; SHR, subdistribution hazard ratio; CI, confidence interval; PPMs, prior primary malignancies.

**Supplementary Figure 6**


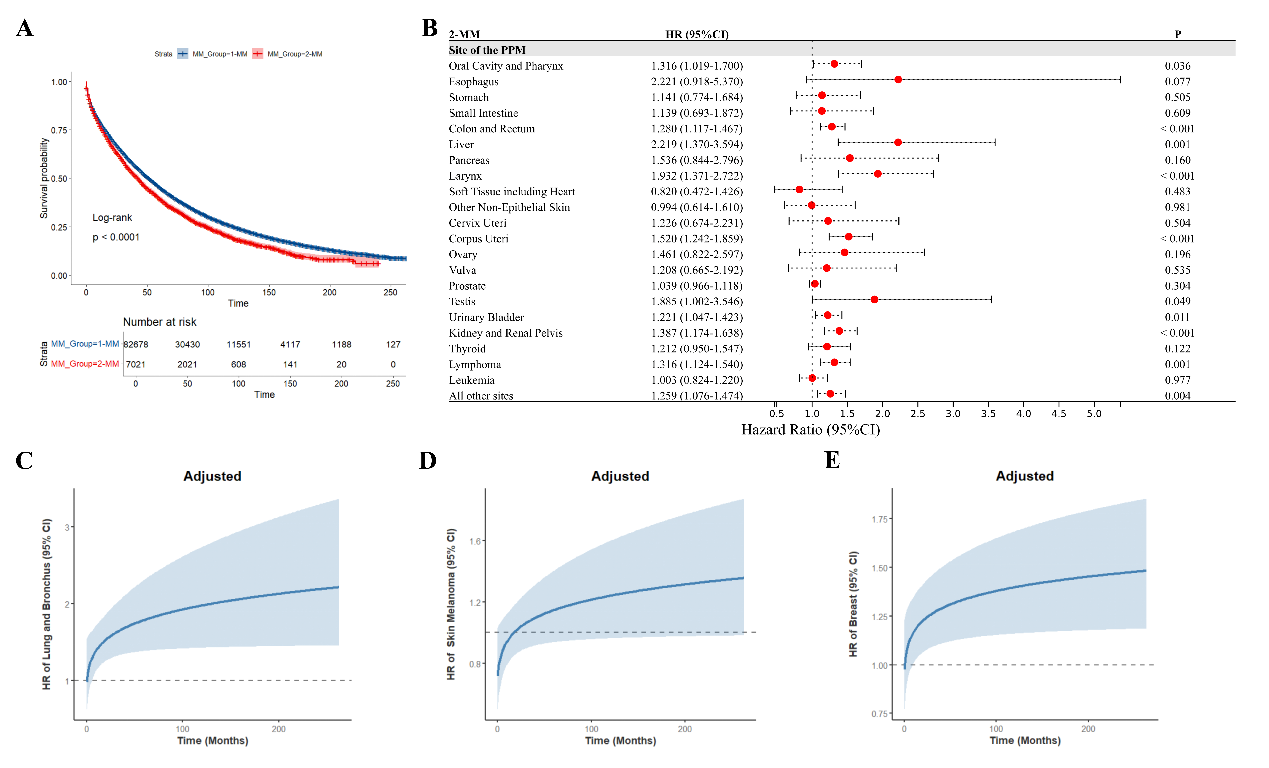


Supplementary Figure 6. Sensitivity analysis of all-cause mortality in the study cohort incorporating all MM patients regardless of subsequent malignancies. (A) Kaplan-Meier curves of overall survival for 2-MM vs. 1-MM. (B-E) Multivariable Cox regression analysis of all-cause mortality (with 1-MM as reference), adjusted for sex, age at diagnosis, race, marital status at diagnosis, year of MM diagnosis, and treatment modalities (chemotherapy, radiotherapy, and surgery) for MM and PPMs. (B) HRs for 2-MM (combined and PPM-stratified subgroups); Time-dependent HRs for PPMs of (C) lung and bronchus, (D) melanoma of the skin, and (E) breast. MM, multiple myeloma; 1-MM, first primary multiple myeloma; 2-MM, second primary multiple myeloma; HR, hazard ratio; CI, confidence interval; PPMs, prior primary malignancies.

**Supplementary Tables**

**Supplementary Table 1.** Clinical characteristics of 2-MM cohort according to PPMs.

| Characteristic | 1-MM  (n = 74932) | 2-MM (n = 6465) | | | | | | | |
| --- | --- | --- | --- | --- | --- | --- | --- | --- | --- |
|  |  | Oral Cavity and Pharynx (n = 104) | Esophagus (n = 10) | Stomach (n = 38) | Small Intestine (n = 30) | Colon and Rectum (n = 550) | Liver  (n = 23) | Pancreas (n=21) | Larynx  (n = 47) |
| Male sex | 54.3% | 78.8%^a^ | 80.0% | 60.5% | 60.0% | 60.0% | 73.9% | 57.1% | 83.0%^a^ |
| Age by category, years |  |  |  |  |  |  |  |  |  |
| 18-54 | 16.9% | 7.7% | 0.0% | 5.3% | 13.3% | 2.7%^a^ | 8.7% | 14.3% | 2.1% |
| 55-64 | 25.0% | 28.8% | 0.0% | 13.2% | 16.7% | 15.6%^a^ | 21.7% | 19.0% | 17.0% |
| 65-74 | 30.0% | 34.6% | 50.0% | 26.3% | 33.3% | 35.3% | 43.5% | 33.3% | 40.4% |
| 75-90+ | 28.0% | 28.8% | 50.0% | 55.3% | 36.7% | 46.4%^a^ | 26.1% | 33.3% | 40.4% |
| Race |  |  |  |  |  |  |  |  |  |
| White | 73.0% | 85.6% | 80.0% | 63.2% | 73.3% | 73.1% | 69.6% | 76.2% | 63.8% |
| Black | 19.8% | 9.6% | 20.0% | 23.7% | 20.0% | 21.8% | 17.4% | 19.0% | 36.2% |
| Asian or Pacific Islander | 6.6% | 1.9% | 0.0% | 13.2% | 6.7% | 4.5% | 13.0% | 4.8% | 0.0% |
| American Indian/Alaska Native | 0.6% | 2.9% | 0.0% | 0.0% | 0.0% | 0.5% | 0.0% | 0.0% | 0.0% |
| Marital status |  |  |  |  |  |  |  |  |  |
| Partnered | 57.0% | 65.4% | 30.0% | 47.4% | 56.7% | 56.7% | 65.2% | 76.2% | 57.4% |
| Previously partnered | 22.9% | 19.2% | 40.0% | 34.2% | 16.7% | 25.5% | 13.0% | 19.0% | 12.8% |
| Single | 13.9% | 9.6% | 10.0% | 7.9% | 10.0% | 11.3% | 13.0% | 4.8% | 23.4% |
| Unknown | 6.3% | 5.8% | 20.0% | 10.5% | 16.7% | 6.5% | 8.7% | 0.0% | 6.4% |
| Diagnosis interval |  |  |  |  |  |  |  |  |  |
| 2000-2009 | 36.0% | 19.2% | 10.0% | 26.3% | 13.3% | 21.1%^a^ | 17.4% | 9.5% | 17.0% |
| 2010-2016 | 33.8% | 35.6% | 0.0% | 28.9% | 33.3% | 41.1% | 39.1% | 38.1% | 44.7% |
| 2017-2021 | 30.2% | 45.2% | 90.0%^a^ | 44.7% | 53.3% | 37.8%^a^ | 43.5% | 52.4% | 38.3% |
| Chemotherapy for MM | 64.8% | 68.3% | 70.0% | 44.7% | 53.3% | 59.1% | 69.6% | 52.4% | 61.7% |
| Radiotherapy for MM | 18.1% | 15.4% | 10.0% | 10.5% | 13.3% | 14.7% | 13.0% | 0.0% | 12.8% |
| Surgery for MM | 0.1% | 0.0% | 0.0% | 0.0% | 0.0% | 0.0% | 0.0% | 0.0% | 0.0% |
| Chemotherapy for PPM | 0.0% | 39.4% | 50.0% | 31.6% | 10.0% | 26.2% | 34.8% | 42.9% | 14.9% |
| Radiotherapy for PPM | 0.0% | 60.6% | 70.0% | 18.4% | 3.3% | 12.7% | 4.3% | 23.8% | 80.9% |
| Surgery for PPM | 0.0% | 64.4% | 50.0% | 89.5% | 90.0% | 96.2% | 73.9% | 71.4% | 51.1% |
| MM specific death | 45.1% | 37.5% | 40.0% | 36.8% | 20.0% | 43.6% | 34.8% | 19.0% | 44.7% |
| All-cause death | 61.6% | 58.7% | 50.0% | 65.8% | 43.3% | 64.5% | 73.9% | 52.4% | 70.2% |

1-MM, first primary multiple myeloma; 2-MM, second primary multiple myeloma; MM, multiple myeloma; PPM, prior primary malignancy.

^a^*P* value less than 0.05 compared with 1-MM.

**Supplementary Table 1.** (Continued 1)

| Characteristic | 1-MM  (n = 74932) | 2-MM (n = 6465) | | | | | | |
| --- | --- | --- | --- | --- | --- | --- | --- | --- |
|  |  | Lung and Bronchus (n = 135) | Soft Tissue including Heart (n = 29) | Melanoma of the Skin (n = 366) | Other Non-Epithelial Skin (n = 29) | Breast  (n = 916) | Cervix Uteri (n = 22) | Corpus Uteri (n = 199) |
| Male sex | 54.3% | 52.6% | 65.5% | 63.1% | 72.4% | 1.2%^a^ | 0.0%^a^ | 0.0%^a^ |
| Age by category, years |  |  |  |  |  |  |  |  |
| 18-54 | 16.9% | 0.7%^a^ | 6.9% | 6.3%^a^ | 3.4% | 4.9%^a^ | 27.3% | 4.5%^a^ |
| 55-64 | 25.0% | 13.3% | 20.7% | 16.1%^a^ | 6.9% | 17.0%^a^ | 22.7% | 14.6% |
| 65-74 | 30.0% | 43.7% | 20.7% | 33.3% | 41.4% | 35.9%^a^ | 27.3% | 41.2% |
| 75-90+ | 28.0% | 42.2% | 51.7% | 44.3%^a^ | 48.3% | 42.1%^a^ | 22.7% | 39.7% |
| Race |  |  |  |  |  |  |  |  |
| White | 73.0% | 68.9% | 75.9% | 100.0%^a^ | 86.2% | 75.8% | 72.7% | 77.4% |
| Black | 19.8% | 21.5% | 17.2% | 0.0%^a^ | 13.8% | 17.2% | 18.2% | 14.6% |
| Asian or Pacific Islander | 6.6% | 8.9% | 3.4% | 0.0%^a^ | 0.0% | 6.1% | 9.1% | 8.0% |
| American Indian/Alaska Native | 0.6% | 0.7% | 3.4% | 0.0% | 0.0% | 0.9% | 0.0% | 0.0% |
| Marital status |  |  |  |  |  |  |  |  |
| Partnered | 57.0% | 60.0% | 82.8% | 66.1% | 51.7% | 48.3%^a^ | 27.3% | 43.2%^a^ |
| Previously partnered | 22.9% | 26.7% | 0.0% | 18.3% | 41.4% | 34.6%^a^ | 50.0% | 39.2%^a^ |
| Single | 13.9% | 7.4% | 10.3% | 11.2% | 6.9% | 9.4%^a^ | 9.1% | 13.1% |
| Unknown | 6.3% | 5.9% | 6.9% | 4.4% | 0.0% | 7.8% | 13.6% | 4.5% |
| Diagnosis interval |  |  |  |  |  |  |  |  |
| 2000-2009 | 36.0% | 17.8%^a^ | 13.8% | 17.5%^a^ | 27.6% | 16.2%^a^ | 9.1% | 17.1%^a^ |
| 2010-2016 | 33.8% | 40.0% | 31.0% | 35.2% | 20.7% | 40.1%^a^ | 36.4% | 37.2% |
| 2017-2021 | 30.2% | 42.2% | 55.2% | 47.3%^a^ | 51.7% | 43.8%^a^ | 54.5% | 45.7%^a^ |
| Chemotherapy for MM | 64.8% | 58.5% | 55.2% | 66.7% | 65.5% | 57.9%^a^ | 68.2% | 62.3% |
| Radiotherapy for MM | 18.1% | 14.1% | 10.3% | 15.8% | 17.2% | 13.1%^a^ | 18.2% | 16.6% |
| Surgery for MM | 0.1% | 0.0% | 0.0% | 0.0% | 0.0% | 0.0% | 0.0% | 0.0% |
| Chemotherapy for PPM | 0.0% | 30.4% | 10.3% | 0.3% | 6.9% | 32.2% | 31.8% | 12.1% |
| Radiotherapy for PPM | 0.0% | 25.9% | 48.3% | 0.3% | 10.3% | 55.9% | 40.9% | 20.1% |
| Surgery for PPM | 0.0% | 70.4% | 100.0% | 97.0% | 89.7% | 97.3% | 72.7% | 98.5% |
| MM specific death | 45.1% | 35.6% | 13.8% | 32.5%^a^ | 34.5% | 37.1%^a^ | 36.4% | 32.7% |
| All-cause death | 61.6% | 72.6% | 41.4% | 50.3%^a^ | 55.2% | 56.0% | 40.9% | 57.3% |

1-MM, first primary multiple myeloma; 2-MM, second primary multiple myeloma; MM, multiple myeloma; PPM, prior primary malignancy.

^a^*P* value less than 0.05 compared with 1-MM.

**Supplementary Table 1.** (Continued 2)

| Characteristic | 1-MM  (n = 74932) | 2-MM (n = 6465) | | | | | | | | | |
| --- | --- | --- | --- | --- | --- | --- | --- | --- | --- | --- | --- |
|  |  | Ovary (n=22) | Vulva  (n = 17) | Prostate  (n = 2474) | Testis  (n = 16) | Urinary Bladder (n = 330) | Kidney and Renal Pelvis (n = 289) | Thyroid  (n = 169) | Lymphoma (n = 254) | Leukemia (n= 150) | All other sites (n = 225) |
| Male sex | 54.3% | 0.0%^a^ | 0.0%^a^ | 100.0%^a^ | 100.0% | 82.1%^a^ | 69.6%^a^ | 36.7%^a^ | 63.4% | 70.7%^a^ | 56.4% |
| Age by category, years |  |  |  |  |  |  |  |  |  |  |  |
| 18-54 | 16.9% | 0.0% | 5.9% | 0.8% | 43.8% | 1.5%^a^ | 8.0%^a^ | 20.7% | 7.5%^a^ | 2.7%^a^ | 5.8%^a^ |
| 55-64 | 25.0% | 18.2% | 35.3% | 11.8%^a^ | 12.5% | 10.3%^a^ | 18.7% | 21.3% | 14.6%^a^ | 14.7% | 14.7% |
| 65-74 | 30.0% | 40.9% | 29.4% | 39.3%^a^ | 18.8% | 29.1% | 37.4% | 34.3% | 31.9% | 40.0% | 30.7% |
| 75-90+ | 28.0% | 40.9% | 29.4% | 48.1%^a^ | 25.0% | 59.1%^a^ | 36.0% | 23.7% | 46.1%^a^ | 42.7%^a^ | 48.9%^a^ |
| Race |  |  |  |  |  |  |  |  |  |  |  |
| White | 73.0% | 81.8% | 64.7% | 70.1% | 87.5% | 87.3%^a^ | 78.9% | 72.2% | 82.7% | 78.0% | 76.9% |
| Black | 19.8% | 13.6% | 35.3% | 26.0%^a^ | 6.2% | 9.7%^a^ | 17.3% | 18.3% | 11.0% | 13.3% | 16.4% |
| Asian or Pacific Islander | 6.6% | 4.5% | 0.0% | 3.5%^a^ | 0.0% | 3.0% | 2.4% | 9.5% | 5.9% | 8.0% | 6.7% |
| American Indian/Alaska Native | 0.6% | 0.0% | 0.0% | 0.4% | 6.2% | 0.0% | 1.4% | 0.0% | 0.4% | 0.7% | 0.0% |
| Marital status |  |  |  |  |  |  |  |  |  |  |  |
| Partnered | 57.0% | 59.1% | 17.6% | 70.3%^a^ | 56.2% | 64.8% | 62.3% | 60.4% | 60.6% | 59.3% | 65.3% |
| Previously partnered | 22.9% | 22.7% | 52.9% | 14.7%^a^ | 6.2% | 21.2% | 21.8% | 24.9% | 20.9% | 22.7% | 20.9% |
| Single | 13.9% | 9.1% | 23.5% | 7.6%^a^ | 18.8% | 7.9% | 10.0% | 8.3% | 10.6% | 11.3% | 9.8% |
| Unknown | 6.3% | 9.1% | 5.9% | 7.4% | 18.8% | 6.1% | 5.9% | 6.5% | 7.9% | 6.7% | 4.0% |
| Diagnosis interval |  |  |  |  |  |  |  |  |  |  |  |
| 2000-2009 | 36.0% | 13.6% | 29.4% | 18.7%^a^ | 12.5% | 17.3%^a^ | 17.0%^a^ | 10.1%^a^ | 18.9%^a^ | 23.3% | 23.6%^a^ |
| 2010-2016 | 33.8% | 27.3% | 23.5% | 42.8%^a^ | 43.8% | 41.5% | 40.5% | 34.3% | 41.7% | 39.3% | 32.4% |
| 2017-2021 | 30.2% | 59.1% | 47.1% | 38.6%^a^ | 43.8% | 41.2%^a^ | 42.6%^a^ | 55.6%^a^ | 39.4% | 37.3% | 44%^a^ |
| Chemotherapy for MM | 64.8% | 72.7% | 70.6% | 60.3%^a^ | 81.2% | 57.3% | 57.1% | 67.5% | 51.6%^a^ | 55.3% | 52.9% |
| Radiotherapy for MM | 18.1% | 27.3% | 11.8% | 16.2% | 37.5% | 14.8% | 15.2% | 14.8% | 9.4% | 12.7% | 9.8% |
| Surgery for MM | 0.1% | 0.0% | 0.0% | 0.0% | 0.0% | 0.0% | 0.0% | 0.0% | 0.0% | 0.0% | 0.0% |
| Chemotherapy for PPM | 0.0% | 54.5% | 11.8% | 0.5% | 12.5% | 16.4% | 2.4% | 0.0% | 53.9% | 30.7% | 31.1% |
| Radiotherapy for PPM | 0.0% | 0.0% | 17.6% | 40.7% | 37.5% | 1.5% | 1.0% | 49.1% | 21.7% | 0.0% | 9.8% |
| Surgery for PPM | 0.0% | 100.0% | 82.4% | 38.6% | 100.0% | 93.3% | 96.9% | 97.0% | 29.5% | 0.0% | 30.2% |
| MM specific death | 45.1% | 31.8% | 47.1% | 39.0%^a^ | 31.2% | 39.1% | 32.5%^a^ | 29.6%^a^ | 31.9%^a^ | 37.3% | 36.0% |
| All-cause death | 61.6% | 54.5% | 58.8% | 61.8% | 62.5% | 63.9% | 60.6% | 41.4% ^a^ | 61.8% | 64.7% | 66.2% |

1-MM, first primary multiple myeloma; 2-MM, second primary multiple myeloma; MM, multiple myeloma; PPM, prior primary malignancy.

^a^*P* value less than 0.05 compared with 1-MM.

**Supplementary Table 2.** Sensitivity analysis of MM-specific and all-cause mortality for PPM treatment modalities in 2-MM cohorts.

| 2-MM | MM-Specific Mortality | | | All-Cause Mortality | | |
| --- | --- | --- | --- | --- | --- | --- |
|  | Adjusted SHR (95% CI) | | | Adjusted HR (95% CI) | | |
|  | Chemotherapy | Radiotherapy | Surgery | Chemotherapy | Radiotherapy | Surgery |
| Combined PPMs (n = 7021) | 1.025 (0.908-1.157) | 1.029 (0.942-1.125) | 0.978 (0.894-1.071) | 1.244 (1.137-1.362)^a^ | 0.939 (0.876-1.007) | 0.862 (0.803-0.925)^a^ |
| Prostate (n = 2669) | - | 0.994 (0.858-1.151) | 0.951 (0.818-1.106) | - | 0.941 (0.840-1.054) | 0.742 (0.656-0.840)^a^ |
| Breast (n = 995) | 0.932 (0.731-1.189) | 0.930 (0.750-1.154) | - | 0.994 (0.818-1.207) | 0.732 (0.617-0.868)^a^ | - |
| Colon and Rectum (n = 595) | 1.135 (0.811-1.590) | 0.975 (0.626-1.519) | - | 1.091 (0.822-1.447) | 1.035 (0.715-1.497) | - |
| Urinary Bladder (n = 362) | 1.216 (0.687-2.152) | - | - | 1.000 (0.642-1.557) | - | - |
| Lymphoma (n = 284) | 1.445 (0.912-2.289) | 0.874 (0.511-1.494) | 0.696 (0.425-1.141) | 1.424 (1.028-1.973)^a^ | 0.998 (0.683-1.457) | 1.036 (0.732-1.468) |
| Corpus Uteri (n = 208) | 0.233 (0.051-1.075) | 1.140 (0.533-2.438) | - | 0.632 (0.315-1.269) | 1.301 (0.782-2.165) | - |

Sensitivity analyses of MM-specific and all-cause mortality for the 2-MM cohorts (combined or PPM-stratified subgroups) regardless of subsequent malignancies were assessed in multivariable models, adjusted for sex, age at diagnosis, race, marital status at diagnosis, year of MM diagnosis, and the other tumor-directed treatment modalities. Specific PPM subgroups were excluded from analyses of particular treatments due to insufficient sample sizes in strata following stratification by PPM treatment modalities. 2-MM, second primary multiple myeloma; MM, multiple myeloma; PPM, prior primary malignancy; SHR, subdistribution hazard ratio; HR, hazard ratio; CI, confidence interval.

^a^*P* value less than 0.05.
